# A Critical Role for Perivascular Cells in Amplifying Vascular Leakage Induced by Dengue Virus Nonstructural Protein 1

**DOI:** 10.1128/mSphere.00258-20

**Published:** 2020-08-05

**Authors:** Yin P. Cheung, Valeria Mastrullo, Davide Maselli, Teemapron Butsabong, Paolo Madeddu, Kevin Maringer, Paola Campagnolo

**Affiliations:** a Department of Biochemical Sciences, Faculty of Health and Medical Sciences, University of Surrey, Guildford, Surrey, United Kingdom; b Department of Microbial Sciences, Faculty of Health and Medical Sciences, University of Surrey, Guildford, Surrey, United Kingdom; c Experimental Cardiovascular Medicine Division, University of Bristol, Bristol Heart Institute, Bristol Royal Infirmary, Bristol, United Kingdom; University of Texas Southwestern Medical Center

**Keywords:** dengue, hemorrhage, perivascular cell, pericyte, NS1, dengue fever

## Abstract

Dengue is the most prevalent arthropod-borne viral disease affecting humans, with severe dengue typified by potentially fatal microvascular leakage and hypovolemic shock. Blood vessels of the microvasculature are composed of a tubular structure of endothelial cells ensheathed by perivascular cells (pericytes). Pericytes support endothelial cell barrier formation and maintenance through paracrine and contact-mediated signaling and are critical to microvascular integrity. Pericyte dysfunction has been linked to vascular leakage in noncommunicable pathologies such as diabetic retinopathy but has never been linked to infection-related vascular leakage. Dengue vascular leakage has been shown to result in part from the direct action of the secreted dengue virus (DENV) nonstructural protein NS1 on endothelial cells. Using primary human vascular cells, we show here that NS1 also causes pericyte dysfunction and that NS1-induced endothelial hyperpermeability is more pronounced in the presence of pericytes. Notably, NS1 specifically disrupted the ability of pericytes to support endothelial cell function in a three-dimensional (3D) microvascular assay, with no effect on pericyte viability or physiology. These effects are mediated at least in part through contact-independent paracrine signals involved in endothelial barrier maintenance by pericytes. We therefore identify a role for pericytes in amplifying NS1-induced microvascular hyperpermeability in severe dengue and thus show that pericytes can play a critical role in the etiology of an infectious vascular leakage syndrome. These findings open new avenues of research for the development of drugs and diagnostic assays for combating infection-induced vascular leakage, such as severe dengue.

**IMPORTANCE** The World Health Organization considers dengue one of the top 10 global public health problems. There is no specific antiviral therapy to treat dengue virus and no way of predicting which patients will develop potentially fatal severe dengue, typified by vascular leakage and circulatory shock. We show here that perivascular cells (pericytes) amplify the vascular leakage-inducing effects of the dengue viral protein NS1 through contact-independent signaling to endothelial cells. While pericytes are known to contribute to noncommunicable vascular leakage, this is the first time these cells have been implicated in the vascular effects of an infectious disease. Our findings could pave the way for new therapies and diagnostics to combat dengue and potentially other infectious vascular leakage syndromes.

## INTRODUCTION

Dengue virus (DENV) is a serocomplex of four viruses (DENV-1 to -4) and the most prevalent arthropod-borne virus (arbovirus) affecting humans. Almost half the world’s population lives in at-risk areas across the tropics and subtropics, with an estimated 390 million infections and 96 million symptomatic cases per year (1). Dengue patients experience a range of symptoms including high fever, leukopenia, maculopapular rash, retro-orbital pain, arthralgia, and myalgia (2, 3). A minority of patients (approximately 500,000 cases per year) develop severe dengue, typified by cardiovascular complications such as plasma leakage (e.g., pleural effusion, edema) and/or bleeding (hemorrhage) manifesting during defervescence that can lead to hypovolemic shock, organ failure, and death (2, 3). Dengue hemorrhage is associated with platelet dysfunction and a consequent thrombocytopenia, and/or disseminated intravascular coagulation (4). Fluid replacement therapy and other supportive treatments reduce the death rate, but there are no specific drugs to treat dengue and no diagnostic measure to predict the development of the life-threatening severe dengue (2–4).

At a microvascular level, the precise mechanisms leading to vascular leakage in severe dengue remain incompletely understood. A dysregulated cytokine response has been proposed to be a major contributor to vascular hyperpermeability, especially in heterotypic secondary infections with a different DENV serotype from the first infection (3, 5, 6). Antibodies against the viral nonstructural protein NS1 have also been shown to bind to endothelial cells in secondary infections, causing their apoptosis (7–9). In addition, NS1 protein is abundantly secreted into patient serum and has been shown to directly induce endothelial cell hyperpermeability (10, 11). Serum NS1 concentrations in dengue patients vary widely. Although concentrations as high as 15,000 ng/ml have been recorded in some patients, concentrations of 10 to 1,000 ng/ml are more typical, with one study suggesting that levels above 600 ng/ml may be predictive of severe disease (12–14). NS1 enters endothelial cells through dynamin- and clathrin-dependent endocytosis and induces the expression of secreted cellular sialidases, heparanase, and cathepsin L that cleave components of the endothelial cell glycocalyx, reducing the integrity of the barrier (15–18). Inhibitors of sialidases and heparanases reduce NS1-dependent endothelial hyperpermeability *in vitro*, and NS1 vaccination has been shown to protect against vascular leakage *in vivo* (11, 17, 19). In addition to these direct effects on the microvascular endothelium, other pathogenic mechanisms contributing to dengue plasma leakage and hemorrhage have also been reported (20), as reviewed in reference 21.

**FIG 1:**
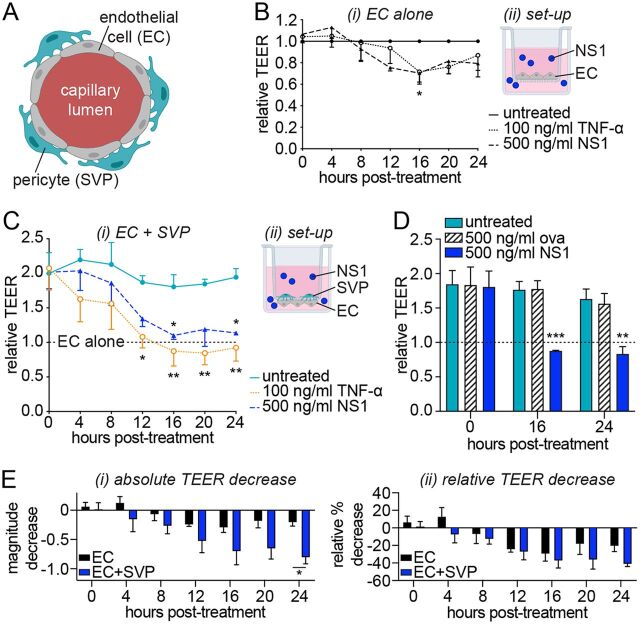
NS1 increases the permeability of an endothelial cell monolayer to a greater degree in the presence of cocultured pericytes. (A) Cross-sectional illustration of the microvascular architecture, with pericytes (SVPs) supporting the maintenance of the endothelial barrier by endothelial cells (ECs). (B) Impact of treatment with purified recombinant TNF-α or DENV-2 NS1 on endothelial cell monolayer permeability over time as measured by transendothelial electrical resistance (TEER). At each individual time point, TEER values are normalized to untreated endothelial cells. (B, ii) Experimental setup for TEER measurements of endothelial cells cultured alone. (C and D) Enhancement of endothelial cell barrier function in coculture with pericytes, and impact of treatment with DENV-2 NS1, TNF-α (C), or ovalbumin (“ova”) (D) on permeability of the coculture over time. At each individual time point, TEER values are normalized to untreated endothelial cells cultured in the absence of pericytes (“EC alone”). (C, ii) Experimental setup of endothelial-pericyte cocultures for data shown in panels C and D. (E) NS1-dependent decrease in TEER for endothelial cells cultured alone or in the presence of pericytes shown as absolute magnitude change in TEER (i) or percent decrease relative to the respective untreated mono- or cocultures (ii). All data, minimum *N* = 3, *n* = 3. Error bars represent standard error of the mean. *, *P *< 0.05; **, *P *< 0.01; ***, *P *< 0.001. In panel B, indicated significance is for NS1 treatment versus untreated endothelial cells. In panels C and D, the difference between “EC alone” and “untreated” (cocultures) is significant at *P* < 0.05. In panel C, significant differences from “untreated” (cocultures) are shown above the line for NS1 treatment and below the line for TNF-α treatment.

The published mechanistic studies into NS1-induced dengue vascular leakage primarily assessed endothelial cell function. However, the vessels affected in severe dengue *in vivo*, primarily capillaries and postcapillary venules, are comprised of an endothelial cell lining surrounded by perivascular cells (pericytes) embedded within the basement membrane (Fig. 1A) (22, 23). There is a dynamic relationship between pericytes and endothelial cells, with two-way paracrine signaling as well as contact-mediated communication facilitated by pericyte pseudopod extensions that wrap around the endothelial cells (24). Pericytes are essential both in blood vessel formation to drive endothelial cell migration, proliferation, and maturation and in homeostasis for the maintenance and regulation of the endothelial barrier in established vessels (25, 26). Pericyte deficiency is perinatally lethal in mouse models due to widespread vascular leakage and aneurysms (27). In established vessels, pericyte dysfunction leads to microvascular hyperpermeability, such as in diabetic retinopathy where chronic exposure to heightened glucose levels due to unmanaged diabetes causes capillary occlusions, microaneurysms, and blindness (28). To date, the role of pericytes in the etiology of vascular hyperpermeability caused by an infectious disease has not been described.

Here, we use microvascular hyperpermeability induced by DENV NS1 as a model to demonstrate a crucial role for pericytes in amplifying an infectious hemorrhagic syndrome. We show that DENV-2 NS1 induces hyperpermeability in *in vitro* cocultures of primary pericytes and primary endothelial cells and that the observed hyperpermeability is greater than that for endothelial cells cultured alone. NS1 does not broadly affect all pericyte functions but rather specifically reduces the capacity of pericytes to support endothelial cell function in three-dimensional (3D) microvascular models. Finally, we demonstrate that NS1-induced hyperpermeability is not dependent on endothelial-pericyte cell contact and is at least partially mediated through effects on paracrine signaling. Our findings could inform new strategies for developing diagnostics and treatments for severe dengue and could have wide-reaching implications for the role of pericytes in other infectious vascular leakage syndromes.

## RESULTS

### NS1 triggers pericyte dysfunction and hyperpermeability in vascular cell cocultures.

We used transendothelial electrical resistance (TEER) as a measure of the strength of a cell monolayer barrier. In line with previous studies (11, 17), we found that treatment with DENV-2 NS1 reduced the barrier function of primary human umbilical vein endothelial cells (HUVECs) starting from 12 h posttreatment (Fig. 1B). The magnitude of this NS1 effect was comparable to that of the vasodilatory cytokine tumor necrosis factor alpha (TNF-α), here used as a positive control (Fig. 1B).

Next, we wanted to explore whether pericytes, which are known to provide fundamental control of microvascular permeability by regulating endothelial cell function (25), contribute to NS1-induced hyperpermeability. Previously, we isolated a population of perivascular cells from the microvasculature surrounding human saphenous veins that expressed bona fide pericyte markers and supported endothelial cell function and angiogenesis *in vitro* and *in vivo* (29). Here, we established an *in vitro* model of the microvascular barrier in which HUVECs were cocultured with saphenous vein pericytes (SVPs) in semicontact, on either side of a porous membrane within a transwell system (Fig. 1C, ii). In this experimental setup, pericytes and endothelial cells interact both via direct cell-to-cell contacts, through the membrane pores (30, 31), and via paracrine signaling mediated by soluble signaling molecules diffusing through the membrane (30, 32). Cells are not able to migrate through the pores of the membranes used in this study. Coculture of endothelial cells with pericytes significantly increased endothelial barrier function compared to endothelial cells cultured alone, thus recapitulating the regulatory function of pericytes (Fig. 1C). Treatment of these endothelial-pericyte cocultures with 500 ng/ml DENV-2 NS1 significantly reduced endothelial barrier function (Fig. 1C). This effect was evident from 8 h posttreatment, and by 16 h the TEER of the cocultures was indistinguishable from that of endothelial cells cultured alone. This indicates that NS1 completely abolished the ability of pericytes to support the barrier function of endothelial cells in this system. In contrast, ovalbumin did not affect permeability of the coculture (Fig. 1D), confirming the specificity of the observed pericyte dysfunction induced by DENV-2 NS1.

Notably, the absolute magnitude of the TEER decrease was significantly higher in the cocultures than in endothelial cells cultured alone, indicating a stronger effect of NS1 on permeability in the presence of pericytes (Fig. 1E, i). Even when we accounted for the higher absolute TEER of the endothelial-pericyte cocultures by normalizing to the respective untreated controls, the decrease in barrier function in the coculture was almost double that of endothelial cell monocultures at 20 h and 24 h posttreatment (Fig. 1E, ii).

Taken together, these results indicate that DENV-2 NS1 induces extensive dysfunction in primary human pericytes, leading to their inability to support endothelial cell barrier function. Furthermore, the effect NS1 has on permeability is more pronounced in the presence of pericytes, indicating that the presence of pericytes amplifies NS1-dependent microvascular hyperpermeability.

### NS1 disrupts the pericyte interaction with and support of the endothelial network.

**FIG 2:**
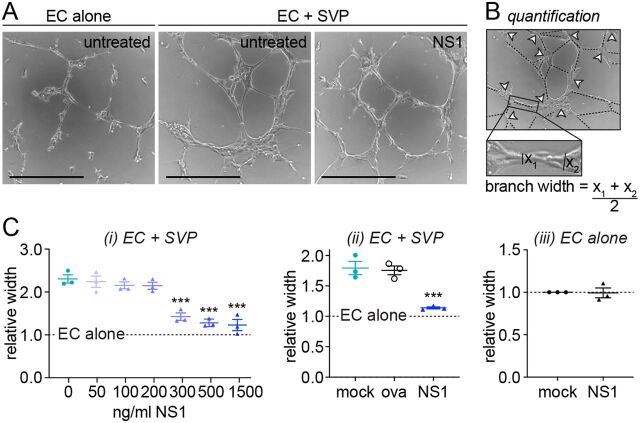
NS1 disrupts pericytes’ ability to support endothelial “capillary” structures in a 3D microvascular model. (A) Representative images of endothelial cells forming capillary-like structures in 3D Matrigel culture in the presence (“EC + SVP”) or absence (“EC alone”) of pericytes, with or without treatment with 500 ng/ml DENV-2 NS1. Bars, 400 μm. (B) Method of quantifying mean width of capillary-like structures in 3D Matrigel cultures using ImageJ. Dashed lines indicate branches (quantified); arrowheads indicate nodes where branches join together (not quantified). Branches were defined as containing elongated capillary-like endothelial cells, while nodes contained only aggregated nonelongated endothelial cells. For each branch, an average of the manual measurements at the thinnest and thickest point was taken. The mean width of all branches across three micrographs taken from one well in a 96-well plate was taken as one technical replicate; biological replicates were from independently set up experiments. (C) Quantification of mean width of capillary-like structures in endothelial-pericyte cocultures (i and ii) or endothelial cells cultured alone (iii) that were treated with NS1 or ovalbumin (“ova”) or left untreated (“mock”). Measurements are normalized to untreated endothelial cells cultured in the absence of pericytes (“EC alone”). Unless specified, recombinant protein concentration was 500 ng/ml. All data, *N* = 3, *n* = 3. Error bars represent standard error of the mean. ***, *P* < 0.001. In panel C, i and ii, significance shown is compared to untreated endothelial-pericyte cocultures; the difference between “EC alone” and untreated cocultures is significant at *P* < 0.0001.

We next wanted to determine whether NS1 acts by disrupting the ability of pericytes to functionally support endothelial cells. Angiogenesis, the formation of new blood vessels from existing ones, is a complex process requiring precisely regulated reciprocal cross talk between endothelial cells, which elongate and migrate to form new lumens, and pericytes, which ensheathe the branches and confer maturity to the new structures (32). Endothelial cells cultured in a 3D Matrigel substrate organize into capillary-like structures, and the maturation of these structures upon coculture with pericytes is an established readout for the ability of pericytes to support endothelial cell and capillary functionality (29). In our hands, endothelial cells cultured on Matrigel formed abundant capillary-like structures after 6 h and the presence of pericytes visibly increased the thickness of the branches of these structures (Fig. 2A). This increase in branch width is due to the physical association of pericytes with the endothelial structures and the secretion of proangiogenic factors by pericytes directly stimulating the endothelium (29).

Treatment of 3D endothelial-pericyte cocultures with 500 ng/ml DENV-2 NS1 caused a reduction in branch width (Fig. 2A), indicating a dysfunctional interaction between endothelial cells and pericytes. Quantification of NS1’s effect revealed a dose-dependent decrease in branch width, with doses above 300 ng/ml completely abolishing the contribution of pericytes to capillary-like structure formation (Fig. 2B and C, i). In contrast, treatment with ovalbumin (“ova”) did not affect the endothelial-pericyte interaction in Matrigel (Fig. 2C, ii), demonstrating the specificity of the NS1 effect. Furthermore, branch width was not impacted by NS1 treatment when endothelial cells were cultured in the absence of pericytes, indicating that NS1 specifically interferes with the ability of pericytes to support the endothelium rather than the intrinsic angiogenic capacity of endothelial cells (Fig. 2C, iii). Taken together, our data suggest that the amplified effect NS1 has on endothelial permeability in the presence of pericytes is due to a disruption of pericyte functionalities required for maintenance of the microvascular architecture.

### NS1 does not broadly disrupt all pericyte functions.

**FIG 3:**
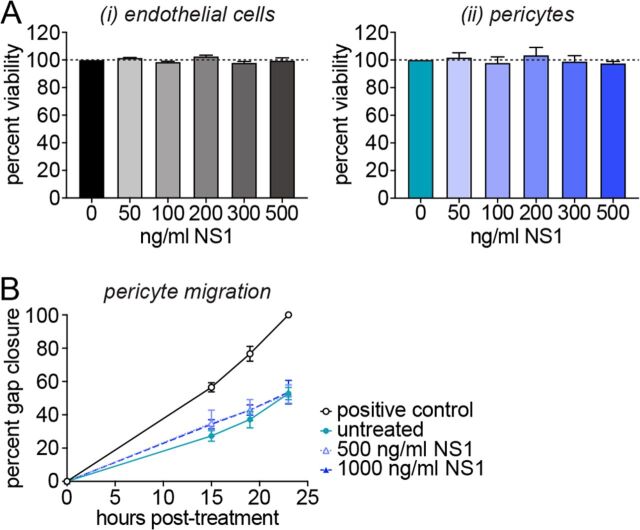
NS1 has no effect on pericyte viability or migration. (A) Impact of treatment with purified recombinant DENV-2 NS1 on the viability of endothelial cells (i) or pericytes (ii) 48 h posttreatment relative to untreated cells. *N* = 4, *n* = 6. (B) Impact of DENV-2 NS1 on the migration of pericytes. All values are normalized to the positive control 23 h postscratch; positive control is pericytes grown in culture medium containing full complement of growth factors. *N* = 3, *n* = 3. Error bars represent standard error of the mean. Differences between treated and untreated cells are not statistically significant.

Our results demonstrate a robust effect of NS1 on pericyte function and in particular on the capacity of pericytes to support endothelial capillary-like structures and barrier function, two defining roles of pericytes. In order to exclude that the observed effect is due to a reduced viability of the pericyte or the endothelial populations, we treated each cell type separately with a range of NS1 concentrations (50 to 500 ng/ml). NS1 treatment for 48 h did not affect cell growth or viability in either endothelial cells or pericytes (Fig. 3A).

In order to support the microvascular architecture, pericytes must be able to migrate along capillaries. For this reason, we tested the intrinsic migratory capacity of pericytes upon treatment with NS1. DENV-2 NS1 concentrations as high as 1,000 ng/ml had no effect on pericyte migration compared to the untreated control in a scratch assay measuring “wound” closure following manual disruption of the cell monolayer (Fig. 3B). These data further support our finding that NS1 specifically affects the endothelial-pericyte interaction.

### NS1 impacts pericyte paracrine signaling that supports the endothelial barrier.

**FIG 4:**
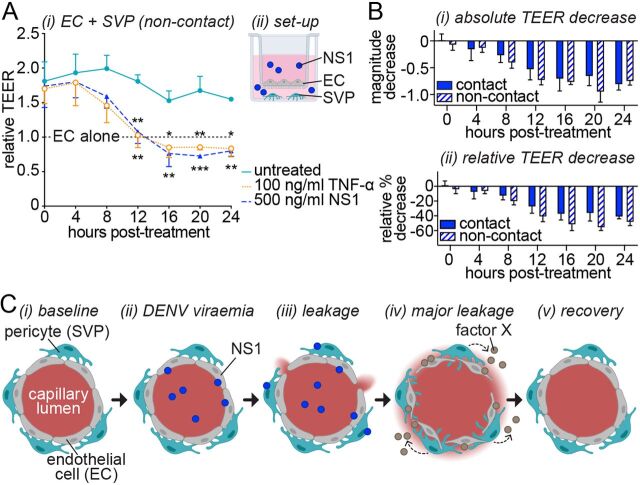
Pericytes amplify NS1-induced endothelial hyperpermeability via disrupted paracrine signaling. (A) Impact of treatment with purified recombinant TNF-α or DENV-2 NS1 on the permeability of endothelial cell monolayers cocultured with pericytes without contact over time as measured by transendothelial electrical resistance (TEER). At each individual time point, TEER values are normalized to untreated endothelial cells cultured in the absence of pericytes (“EC alone”). (A, ii) Experimental setup of endothelial-pericyte cocultures for data shown in panel A. (B) NS1-dependent decrease in TEER for endothelial-pericyte cocultures grown with or without contact shown as absolute magnitude change in TEER (i) or percent decrease relative to the respective untreated cocultures (ii). (C) Working model for mechanism of pericyte amplification of NS1-induced endothelial permeability. (i) Under baseline conditions, pericytes support barrier maintenance by endothelial cells in the microvasculature. (ii and iii) During DENV-2 infection (ii), NS1 secreted into the circulatory system directly targets endothelial cells to (iii) induce vascular leakage, allowing NS1 to access pericytes as NS1 levels peak. (iv) Pericyte function is modulated by NS1, causing pericytes to secrete a paracrine factor (“factor X”) that signals to endothelial cells to further disrupt the endothelial barrier, resulting in enhanced vascular leakage after NS1 levels peak. (v) Pericytes recover from transient NS1 effects, and normal endothelial-pericyte signaling is restored to allow cardiovascular recovery. All data, *N* = 3, *n* = 4. Error bars represent standard error of the mean. *, *P* < 0.05; **, *P* < 0.01; ***, *P* < 0.001. In panel A, the difference between “EC alone” and “untreated” (cocultures) is significant at *P* < 0.05; significant differences from “untreated” (cocultures) are shown above the line for TNF-α treatment and below the line for NS1 treatment.

Pericytes regulate endothelial permeability through cell-cell interactions and paracrine signaling (25). In order to assess the paracrine contribution of pericytes to endothelial barrier function, we modified our TEER assay by seeding pericytes on the bottom of the well instead of on the membrane. This prevents any contact between endothelial cells and pericytes across the porous membrane. In this noncontact TEER, coculture of endothelial cells with pericytes increased endothelial barrier function to a similar extent as that observed in semicontact TEER (Fig. 4A, compared to Fig. 1C). This confirms that pericytes’ contribution to the capillary barrier is not simply as an additional physical layer but as a master regulator of endothelial permeability. Treatment with 500 ng/ml DENV-2 NS1 decreased the resistance of the endothelial cell monolayer in the noncontact coculture starting at 8 h posttreatment, and from 12 h posttreatment the contribution of pericytes to endothelial barrier function was completely ablated (Fig. 4A). Furthermore, neither the absolute nor the relative effect size of NS1 treatment was significantly different comparing the contact and noncontact cocultures (Fig. 4B), indicating that the effect of NS1 is mediated at least in part through paracrine modulators of endothelial barrier function.

## DISCUSSION

Here, we have described a role for pericytes in amplifying the hyperpermeability-inducing effects of DENV-2 NS1 on endothelial cells *in vitro* and in doing so demonstrated that pericytes play a crucial role in the etiology of an infectious hemorrhagic syndrome. NS1 had no effect on pericyte viability or migration, suggesting that NS1 specifically reduces the capacity of pericytes to support endothelial cell functions required for microvascular barrier formation and maintenance. We furthermore demonstrated that the effects of NS1 are at least partially the result of altered paracrine signaling between pericytes and endothelial cells. Since pericytes are an essential component of the microvascular architecture, we propose that pericytes play a crucial role in the etiology of severe dengue. Interestingly, the distantly related flavivirus Japanese encephalitis virus (JEV) was recently shown to infect pericytes in the blood-brain barrier, where pericytes are also fundamental in maintaining the integrity of the barrier, allowing the virus to gain access to the brain to cause encephalitis (33). Pericytes may therefore play a wider role in the pathogenesis of flaviviruses that lead to diverse symptomatic outcomes.

Our data strongly suggest that NS1 interferes with the dynamic cross talk between endothelial cells and pericytes. The specific molecular mechanisms and factors mediating the NS1-dependent hyperpermeability remain unknown. For example, N-glycosylation of NS1 at position 207 is required for endocytosis into endothelial cells and hence the subsequent degradation of the glycocalyx that results in endothelial cell permeability (15). Whether NS1 glycosylation is similarly important for the interaction between NS1 and pericytes is not known and would be interesting to investigate further. On the host side, among several vasoactive molecules reported to be overrepresented in dengue patients, vascular endothelial growth factor (VEGF) has been shown to be upregulated 20-fold compared to healthy controls (6). VEGF is abundantly (but not exclusively) secreted by pericytes to control endothelial cell angiogenesis and negatively regulates pericyte function (34, 35). Platelet-derived growth factor BB (PDGF-BB), a critical regulator of pericyte association with the endothelium, was also upregulated 20- to 60-fold in dengue patients, with a further increase at defervescence in patients with severe dengue (36). This timing coincides with the development of severe symptoms and is suggestive of an endothelial response to pericyte dysfunction. Finally, low levels of angiopoietin 1 (Ang1) and elevated levels of its antagonist angiopoietin 2 (Ang2) have been associated with dengue vascular leakage (5, 37), and the Ang1/Ang2 ratio has been proposed as a diagnostic marker for patients at risk of developing severe dengue (38). Ang1 is secreted by pericytes and platelets to increase endothelial barrier function (32), while Ang2, produced by endothelial cells and affecting pericyte coverage, is known to cause pericyte dropout in diabetic retinopathy (32, 39). It is tempting to speculate that one or more of these factors clinically associated with severe dengue might be indicative of a perturbation of pericyte function by NS1 *in vivo*. Exploring these various potential mechanisms underlying our observed effects of NS1 on the endothelial-pericyte interaction will provide fruitful avenues for future investigation.

We propose a mechanism whereby, early during DENV infection, NS1 secreted into patient serum induces well-described direct effects on endothelial cells causing local permeability that allow NS1 to gain access to pericytes on the apical side of the endothelium (Fig. 4C, i to iii). Subsequent effects of NS1 on pericytes cause a dysregulation of paracrine signaling between pericytes and endothelial cells that leads to pronounced and potentially life-threatening hyperpermeability that manifests during defervescence (Fig. 4C, iv). In patients who recover, the integrity of the microvascular endothelium is restored as pericytes regain the ability to support endothelial cell functions crucial to endothelial barrier formation and maintenance (Fig. 4C, v). Indeed, our experimental setup specifically mimics a scenario in which NS1 targets both endothelial cells and pericytes concurrently following permeabilization of the endothelial barrier, since we added NS1 to both sides of the transwell membrane (Fig. 1C, ii, and Fig. 4A, ii). It is also feasible that NS1, which is endocytosed by endothelial cells (15), may additionally access pericytes via transcytosis through endothelial cells prior to vascular permeabilization *in vivo*; however, this hypothesis remains to be tested. It seems unlikely that NS1 can target pericytes *in vivo* without first impacting on endothelial cells given that the major sites of DENV replication (and hence NS1 production) are monocyte-derived immune cells that secrete NS1 directly into the circulation. It is also feasible that pericyte function is indirectly disrupted due to a perturbation of endothelial cell signaling to pericytes by NS1, and this remains to be investigated.

Concentrations as high as 15,000 ng/ml of NS1 have been observed in the serum of dengue patients; however, patients with severe dengue most commonly have serum NS1 levels ranging between 10 and 1,000 ng/ml (12–14). In our hands, NS1 affected the ability of pericytes to support endothelial cell function in 3D microvascular cocultures at concentrations as low as 300 ng/ml (Fig. 2C). To our knowledge, this is the lowest concentration at which microvascular effects of NS1 have been demonstrated *in vitro*. Furthermore, in endothelial-pericyte cocultures, the observed reduction in TEER upon treatment with 500 ng/ml of NS1 is dramatically larger than that in endothelial cells cultured alone (Fig. 1E). We therefore propose that modeling the essential role pericytes play in maintaining the microvascular endothelial barrier improves the ability of *in vitro* models of dengue hyperpermeability to recapitulate the effects of NS1 at more patient-relevant NS1 concentrations. Studying the effects of NS1 in our improved endothelial-pericyte coculture system may identify novel molecular pathways and cellular mechanisms of dengue microvascular leakage that could facilitate the development of new diagnostics and treatments for severe dengue. Furthermore, our findings pave the way for future studies into the contribution that pericytes may make to the pathogenesis of other infectious diseases manifesting with symptoms of microvascular leakage.

## MATERIALS AND METHODS

### Cell culture.

Human umbilical vein endothelial cells (HUVECs) were purchased from Lonza (Basel, Switzerland). Saphenous vein pericytes (SVPs) were isolated from patient-derived saphenous vein adventitial vasa vasorum by enzymatic digestion using CD34^+^ CD31^−^ selection as described in our previous publication (29). Both cell types were cultured in endothelial growth medium 2 (EGM-2; PromoCell, Heidelberg, Germany) and maintained at 37°C with 5% CO_2_. SVPs were grown on 1% fibronectin and 0.05% (vol/vol) gelatin-coated flasks. Cells were grown to 80% confluence and then passaged and used up to passage 8 for experiments.

### Recombinant proteins.

Human embryonic kidney 293 (HEK293)-produced DENV-2 NS1 was purchased from the Native Antigen Company (Kidlington, United Kingdom). Recombinant NS1 used between 50 and 1,500 ng/ml as indicated in the figures and figure legends. Ovalbumin (Invivogen, San Diego, CA, USA) was used as negative control at 500 ng/ml, and TNF-α (Peprotech, Rocky Hill, NJ, USA) was used as a positive control at 100 ng/ml.

### Matrigel angiogenesis assay.

Each well of a 96-well plate was coated with 30 μl of growth factor-reduced Matrigel (Corning, Corning, NY, USA) on ice and allowed to solidify at 37°C for 30 min. Single cultures of 2 × 10^4^ cells/well of HUVECs or cocultures of 1.5 × 10^4^ cells/well of HUVECs and 0.5 × 10^4^ cells/well of SVPs were seeded on top of the Matrigel. Three bright-field images were taken from each well 6 h after treatment.

### TEER.

Millicell transwell inserts for 24-well plates (0.4-μm pores; Merck Millipore, Watford, United Kingdom) were coated with 1% fibronectin on both sides. Single cultures were established by seeding 1 × 10^5^ HUVECs per insert on one side of the transwell. For contact cocultures, 2.5 × 10^4^ SVPs per insert were seeded on the other side of the transwell, 24 h later. For noncontact cocultures, SVPs were alternatively seeded at the bottom of the 24-well plate. Cells were cultured to confluence, and 50% of the medium was changed every 48 h. Cells were treated by removing 50% of the culture medium from both the well and the transwell insert and making up the volume with fresh medium containing recombinant NS1 or control proteins. Concentrations shown in figures are final concentrations. Transendothelial electrical resistance (TEER) readings were taken using a Millicell ERS-2 voltohmmeter (Merck Millipore) immediately after treatment (0 h) and every 4 h for 24 h thereafter. Absolute TEER was calculated by subtracting the blank and multiplying by the well area; relative TEER was derived by dividing all values by the value of the HUVEC monocultures at each time point.

### Migration assay (wound healing).

SVPs were seeded in 48-well plates and allowed to grow to confluence, at which point the monolayer was scratched using a 10-μl pipette tip. The culture medium was changed to remove detached cells and treatments as indicated in endothelial basal medium (EBM; PromoCell) in the presence of 2 mM hydroxyurea to prevent cell proliferation. Pictures of the scratches were taken in the same positions every 4 h. Gap closure was calculated as the percentage of the distance traveled by the cell front divided by the initial size of the gap and normalized to the positive control within each replicate. The positive control was pericytes grown in culture medium containing a full complement of growth factors.

### Cell viability assay.

HUVECs and SVPs were seeded in 96-well plates at 7 × 10^3^ cells/well and 2 × 10^3^ cells/well, respectively. After overnight adhesion, cells were treated with recombinant proteins for 24 or 48 h. AlamarBlue (ThermoFisher Scientific, Waltham, MA, USA) was then added 1:10 into the culture medium, and fluorescence was measured after 3 h using a spectrophotometer (excitation, 545 nm; emission, 584 nm). Relative viability was calculated in relation to the negative control.

### Microscopy and image analysis.

Images were captured using either an Eclipse Ts2 (Nikon, Tokyo, Japan) or an EVOS cell imaging system (ThermoFisher) inverted microscope. All images for a single analysis were collected using the same settings. For 3D microvascular cultures in Matrigel, branch width values were calculated manually using Fiji (ImageJ) image analysis software (National Institutes of Health, Bethesda, MD, USA) (Fig. 2B). For each branch an average of the manual measurements at the thinnest and thickest point was taken. The mean width of all branches observed in the three micrographs from a single well of the 96-well plate was taken as one technical replicate; biological replicates were from independently set up experiments. On average, approximately 100 branches were measured per condition for each experiment. To avoid bias, a subset of images from each experiment was blinded and independently verified by another lab member. All width measurement data are minimum *N* = 3, *n* = 3.

### Statistical analysis.

Values are reported as mean ± SEM. Experiments were repeated with a minimum of three independent biological replicates and three technical repeats within each replicate. Statistical analyses were performed with GraphPad Prism 8 software (GraphPad Software Inc., San Diego, CA, USA). For Fig. 1B and C, Fig. 3B, and Fig. 4A, statistical analysis was by two-way repeated-measures analysis of variance (ANOVA) with Dunnett correction for multiple comparisons. Figure 1D and E and Fig. 4B were analyzed by two-way repeated-measures ANOVA with Sidak correction for multiple comparisons. Figure 2C, i and ii, and Fig. 3A were analyzed by one-way ANOVA with Dunnett correction for multiple comparisons. Figure 2C, iii, was analyzed by unpaired Student’s *t* test.

### Figures.

Graphs were plotted in GraphPad Prism 8 software. Illustrations were created with BioRender.com (Toronto, ON, Canada). Figures were prepared in Adobe Illustrator (Adobe Systems, San Jose, CA, USA). Images were cropped, annotated, and modified to optimize brightness and contrast only.
